# A Rasch and confirmatory factor analysis of the General Health Questionnaire (GHQ) - 12

**DOI:** 10.1186/1477-7525-8-45

**Published:** 2010-04-30

**Authors:** Adam B Smith, Lesley J Fallowfield, Dan P Stark, Galina Velikova, Valerie Jenkins

**Affiliations:** 1 Centre for Health & Social Care, Charles Thackrah Building, University of Leeds, UK; 2 Psychosocial Oncology Group Cancer Research UK, University of Sussex, Brighton, UK; 3 Cancer Research UK - Clinical Centre, St. James's Institute of Oncology, St. James's University Hospital, Leeds, UK

## Abstract

**Background:**

The General Health Questionnaire (GHQ) - 12 was designed as a short questionnaire to assess psychiatric morbidity. Despite the fact that studies have suggested a number of competing multidimensional factor structures, it continues to be largely used as a unidimensional instrument. This may have an impact on the identification of psychiatric morbidity in target populations. The aim of this study was to explore the dimensionality of the GHQ-12 and to evaluate a number of alternative models for the instrument.

**Methods:**

The data were drawn from a large heterogeneous sample of cancer patients. The Partial Credit Model (Rasch) was applied to the 12-item GHQ. Item misfit (infit mean square ≥ 1.3) was identified, misfitting items removed and unidimensionality and differential item functioning (age, gender, and treatment aims) were assessed. The factor structures of the various alternative models proposed in the literature were explored and optimum model fit evaluated using Confirmatory Factor Analysis.

**Results:**

The Rasch analysis of the 12-item GHQ identified six misfitting items. Removal of these items produced a six-item instrument which was not unidimensional. The Rasch analysis of an 8-item GHQ demonstrated two unidimensional structures corresponding to Anxiety/Depression and Social Dysfunction. No significant differential item functioning was observed by age, gender and treatment aims for the six- and eight-item GHQ. Two models competed for best fit from the confirmatory factor analysis, namely the GHQ-8 and Hankin's (2008) unidimensional model, however, the GHQ-8 produced the best overall fit statistics.

**Conclusions:**

The results are consistent with the evidence that the GHQ-12 is a multi-dimensional instrument. Use of the summated scores for the GHQ-12 could potentially lead to an incorrect assessment of patients' psychiatric morbidity. Further evaluation of the GHQ-12 with different target populations is warranted.

## Background

The General Health Questionnaire belongs to a family of instruments for assessing psychiatric morbidity in both community and non-psychiatric settings [1]. The original General Health Questionnaire (GHQ) comprised 60 items and versions with fewer items have been developed from this, e.g. the GHQ - 30, GHQ - 28 and GHQ- 12 [1,2]. The GHQ -12 is a brief, well validated instrument [3], yet despite its brevity there has been considerable debate in the literature regarding the dimensionality of the instrument. Although originally intended as a unidimensional instrument, a number of exploratory and confirmatory factor analysis studies have found evidence for two- and three factor structures.

Politi et al. [4] used a principal components analysis to explore the dimensionality of the GHQ - 12 and identified a two factor structure corresponding to a seven-item "General Dysphoria" factor consisting of the anxiety and depression items, and a six-item "Social Dysfunction" function, consisting of items relating to daily activities and ability to cope. One item (item 12, "Not feeling happy") loaded weakly onto both factors. Similarly, others [5] have found evidence of two structures (Anxiety/Depression and Social Dysfunction with seven and five items respectively) closely resembling that proposed by Politi et al. [4].

An alternative two factor model has also been proposed [6] consisting of a six-item Anxiety/Depression factor and a five-item Daily Activities and Social Performance factor with one item ("could not concentrate") not loading onto either of these factors. Other two factor models have been reported in the literature [7], the most significant of which has been derived from the World Health Organization's study of psychological disorders in 15 international general health care centres [3], which found evidence for a Depression (4 items) and a Social Dysfunction (3 items) factor.

In addition to these a number of three factor models have also been suggested [8,9]. There is some evidence [10] to support the model proposed by Worsley and Gribbin [11] consisting of three factors ("Social Performance", "Anhedonia" and "Loss of confidence") with three cross-loading items (e.g. "concentrate", "enjoy normal activities", and "feeling reasonably happy"), although a significant number of population-based studies have provided support for Graetz's [12] three factor model comprising Anxiety/Depression, Social Dysfunction and Loss of Confidence [13-17].

Finally, a recent study [18] using confirmatory factor analysis, where poorly performing items were removed on the basis of the squared multiple correlations, found support for an eight-item GHQ corresponding to a 4-item (positively worded) "Social Dysfunction" factor, and a four-item (negatively worded) "Anxiety and Depression". This particular study employed six response categories (ranging from 0 = "never" to 5 = "all the time") rather than the usual four categories used for the GHQ-12 (see below).

Despite the various two- and three- factor models proposed the high degree of correlation reported between factors has often led a number of authors to recommend using the summed GHQ - 12 scores [14,15,19], yet the factor structure has important implications on the reliability and validity of the instrument, as well as on interpreting scores [20] and how the GHQ-12 should be used to identify psychiatric morbidity. Traditional psychometric methods have been unable to provide a definitive answer, however modern psychometric models have shed further light on the dimensionality of the GHQ. A Rasch analysis of the GHQ-28 [21] has revealed a two factor structure based on positive and negatively worded items. Indeed a number of the factor structures proposed for the GHQ-12 have demonstrated separate factor loadings based on valence of the items [12,18]. A recent study has suggested that the putative models proposed for the GHQ-12 may, in fact, be an artefact caused by a response bias to the negative wording of six of the items [22]. This study assessed the dimensionality of the GHQ-12 using confirmatory analysis allowing error terms on the negatively worded items to correlate. The results provided evidence for a GHQ-12 unidimensional structure when response bias was taken into consideration.

However, no analysis of the GHQ - 12 has been undertaken to date using non-sample dependent models, such as Rasch Models.

The aim of this study was to explore the dimensionality of the GHQ12 using Rasch models, in particular to ascertain whether the GHQ12 is a unidimensional structure. The secondary aim was to evaluate the dimensionality of the GHQ -8 using a Rasch analysis and furthermore to assess any resultant factor structure of the GHQ-12 and GHQ-8 using Confirmatory Factor Analysis in comparison with some of the previously proposed models.

## Methods

### Patients

A total of 2934 cancer patients (females = 1718 and males = 1086) with heterogeneous diagnoses completed the GHQ12. The main diagnoses were breast cancer 27%, gastro-intestinal 18%, lymphomas and haematological cancers 8%, lung 7%, and gynaecological 7%. In addition to malignant cancers a small number of patients (144/2934, 5%) had a diagnosis of non-malignant cancer. Details were also available regarding treatment aims (curative 41%, palliative 36.5%, remission 10%, as well as uncertain, missing or not applicable 12.5%). Data regarding patient age was available for 2804 patients. The average age of these patients was 57.42 years (females = 56.96, males = 58.12). The patients were recruited from several studies conducted by the Cancer Research UK Psychosocial Oncology Group, Brighton & Sussex Medical School, UK. The studies from which the data were drawn have all received local ethics approval. Further patient details have been published elsewhere [23-25].

### Instrument

The GHQ12 is a 12-item instrument designed for assessing and detecting psychiatric morbidity [2]. There are four response categories for each item, i.e. "Better than usual", "Same as usual", "Less than usual" and "Much less than usual". Six of the items are positively worded; the other six are negatively worded. Along with the original dichotomous scoring system (0-0-1-1), a modified dichotomous system (0-1-1-1) has also been advocated to identify individuals with existing psychiatric morbidity [26]. Finally, the GHQ12 may also be scored as a Likert scale (on a 0-3 scale). There is evidence to suggest that ordinal, Likert scoring of the GHQ-12 allows better discrimination between competing models in confirmatory factor analyses of the GHQ-12 [27]. Given the various scoring methods recommended for the GHQ-12 an initial Rasch analysis was carried out on the instrument to determine whether the ordinal, Likert scoring was appropriate for the data (described in detail below).

### Rasch Analysis

Rasch models [28] are latent trait models estimating person ability (or person measure), and item difficulty along a single continuum. Rasch Models describe a probabilistic relationship between item difficulty and person ability both of which are reported in "logits" or log-odds. In addition to this, thresholds are derived for each adjacent response category in a scale and each threshold has its own estimate of difficulty. Distances between thresholds should increase monotonically, that is, the average person ability required to endorse individual categories should increase across categories. Ordered categories would support a polytomous scoring system (e.g. Likert) for instruments (e.g. GHQ-12), whereas disordered thresholds would indicate that categories may need to be collapsed.

There are two other important criteria for Rasch Models, namely item fit and dimensionality. Item fit to the Rasch model is commonly measured by the mean-square residual fit statistic [29]. Two commonly employed fit statistics to assess item fit are the weighted mean square or infit statistic, and the unweighted mean square or outfit statistics. The outfit statistic is sensitive to anomalous outliers for either person or item parameters, whereas the infit statistic is sensitive to residuals close to the estimated person abilities. Fit statistics for items have an expected value of 1.0, and can range from 0 to infinity. Deviations in excess of the expected value can be interpreted as 'noise' or lack of fit between the items and the model, whereas values significantly lower than the expected value can be interpreted as item redundancy or overlap.

Dimensionality concerns whether the data form a single factor [29] and can be used to assess whether the single latent trait explains all the variance in the data, i.e. whether the instrument is unidimensional. Dimensionality may be evaluated using principal components analyses (PCA) of the residuals once the initial latent trait (i.e. the "Rasch" factor) has been extracted [29]. Any potential multidimensionality identified by the PCA can be investigated further using a method described by Smith [30].

The final issue to consider is item invariance. Rasch models require item estimation to be independent of the subgroups of individuals completing the questionnaires. In other words, item parameters should be invariant across populations [29]. Items not demonstrating invariance are referred to as demonstrating differential item functioning (DIF). A DIF analysis assesses whether items are functioning equivalently across important categories, such as diagnosis, and extent of disease.

### Rasch Analysis

Details of the application of Rasch Models to mental health instruments can be found in a number of publications [31,32]. A Rasch model (Partial Credit Model) for polytomous data [33] was used to analyse the data using *Winsteps *software [34].

### Analysis of the GHQ-12

#### Item thresholds

Distances between item thresholds were derived and evaluated for threshold disordering.

#### Item Fit

Item fit was evaluated iteratively and misfitting items (mean square infit statistics ≥ 1.3) removed. The remaining items were then recalibrated and fit re-evaluated until no further misfit was observed.

#### Dimensionality

Dimensionality of the GHQ-12 was assessed using a principal components analysis of the residuals. Percentage variance explained in excess of 60% and eigenvalues greater than 3 was taken as initial evidence of unidimensionality [34]. In addition, Smith's method [30] was employed to further identify any multidimensionality: Item parameters for misfitting items were estimated with the entire scale, as well as independently for the misfitting items alone. These two estimates for each misfitting item were then subtracted from each other and an average, or shift constant [30] calculated. Person measures were calculated for the entire scale (including misfitting items), as well as using the misfitting items alone. The latter were then weighted using the shift constant (added to the person measures estimated by the misfit items alone) and independent t-tests performed for each pair of person measures. The percentage of tests falling outside the 95% confidence interval, + 1.96, may then be evaluated. Any significant number of tests outside this interval would indicate the presence of multidimensionality.

#### Differential Item Functioning

Differential item functioning (DIF) was investigated for gender, treatment aims (four categories: curative, remission, palliative and uncertain/missing) and age group (three categories based on tertiles: < = 51; > 51 & < = 63; and > 63 years of age) by estimating item locations for each subgroup and evaluating these using paired t-tests [34] (Linacre, 2008). A minimum difference in scores of 0.5 logits was employed to overcome the problem of multiple testing [35].

#### Rasch Analysis of the GHQ-8

A separate Rasch analysis was undertaken for each of the two GHQ-8 factors (Social Dysfunction, and Anxiety and Depression) using the same methodology as described above for the GHQ-12.

#### Confirmatory Factor Analysis

**Figure 1:**
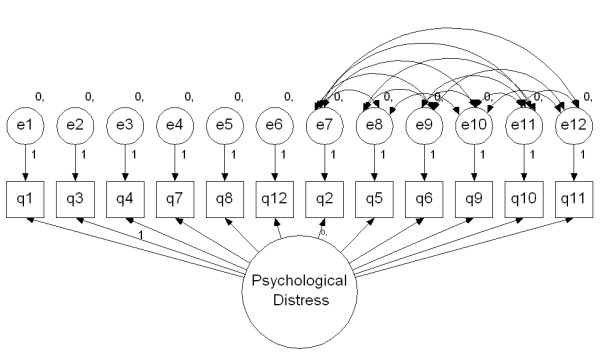
**GHQ-12 Hankins' (2008) Single factor model with correlated error terms**.

The various proposed factor structures for the GHQ-12, including the Rasch construct and the GHQ-8 were tested using confirmatory factor analysis (CFA) in *AMOS 7 *(SPSS version 15). An additional version of the single factor model (Figure 1) was assessed by modelling correlated error terms for the negatively worded items [22].

Maximum likelihood estimation was used for the CFA. The goodness-of-fit of each model was assessed using the Sattora-Bentler scaled chi-square, the comparative fit index [36] (CFI) and the incremental fit index [37] (IFI). Additionally, the root-mean-square error of approximation [38] (RMSEA) was included with 90% confidence intervals. Non-significant chi-squares and values greater than 0.95 are considered as acceptable model fit for the CFI and IFI. RMSEA values below 0.08 are considered to reflect acceptable fit to the model and values smaller than 0.05 as good fit [39]. Finally, a comparison of fit between the various models was also included using the expected cross-validation index [40] (ECVI). The smallest value for the ECVI was used to indicate the best model fit [15].

## Results

A summary of each model assessed is shown in Table 1.

**Table 1:** A summary of the five GHQ models

Items	GHQ-12	Two factor^1^		Three factor^2^			GHQ-8^3^		GHQ-6
		**Positive**	**Negative**	**Social Dysfunction**	**Anxiety/Depression**	**Confidence**	**Social Dysfunction**	**Anxiety/Depression**	

Been able to concentrate	*	*		*					
Lost much sleep over worry	*		*		*				
Felt that you are playing a useful part	*	*		*					
Felt capable of making decisions	*	*		*			*		
Felt constantly under strain	*		*		*				*
Felt you couldn't overcome your difficulties	*		*		*			*	*
Been able to enjoy your normal activities	*	*		*			*		
Been able to face up to your problems	*	*		*			*		*
Been feeling unhappy and depressed	*		*		*			*	*
Been losing confidence in yourself	*		*			*		*	*
Been thinking of yourself as worthless	*		*			*		*	
Been feeling reasonably happy	*	*		*			*		*

^1^Andrich & van Schoubroeck (1989)

^2^Graetz (1991)

^3^Kalliath et al. (2004)

### Item summaries

The item summary is shown in Table 2. It can be seen that item means were lower in general for negatively worded items suggesting these items were harder to endorse. These results are similar to those from an earlier Rasch analysis of the GHQ-28 [21]. Furthermore, similar to other findings [22] item variance was greater for negatively worded items than positively worded items.

**Table 2:** Item means, variance and distance between item thresholds for the GHQ-12

Item:	Mean	Variance	Distance between item thresholds:		
**Positive**			**1**	**2**	**3**

Q1	1.37	0.438	-4.37	5.20	2.71
Q3	1.28	0.463	-4.05	5.09	1.97
Q4	1.14	0.287	-4.92	6.39	1.98
Q7	1.51	0.566	-3.73	4.37	2.45
Q8	1.12	0.277	-4.91	6.31	2.11
Q12	1.20	0.405	-4.23	5.45	1.79
					

**Negative**					

Q2	1.00	0.686	-2.60	2.90	2.00
Q5	1.22	0.650	-2.91	2.87	2.99
Q6	0.95	0.546	-3.11	3.61	2.12
Q9	1.12	0.790	-2.29	2.09	2.69
Q10	0.84	0.696	-2.28	2.27	2.30
Q11	0.24	0.492	2.46	-6.11	4.84
Q11*	0.14	0.166	-1.28	2.56	

### Rasch Analysis of the GHQ-12

#### 1. Item thresholds

Distances between item thresholds are shown in Table 2. It can be seen that item 11 ("Been thinking of yourself as a worthless person") was the only item to display threshold disordering, i.e. between the second and third category ("No more than usual" and "Rather more than usual"). These two categories were subsequently collapsed into a single category for this item, which revealed no further disordering on a subsequent re-analysis (identified in Table 2 as "Q11*").

The lack of threshold disordering supports the use of the Likert scoring method for the GHQ-12 as opposed to the dichotomous scoring method. Therefore, the former scoring method was used throughout for the subsequent analyses (with a three-point, rather than 4-point Likert scale applied to item 11).

The range of thresholds was smaller for the negatively worded items in comparison with the positively worded questions. This result mirrors that of Andrich and van Schoubroek's [21] analysis of the GHQ-28, and in addition to suggesting that the negatively and positively worded items are functioning differently, it also implies that negatively worded items discriminate better than positively worded items.

#### 2. Item Fit GHQ-12

A total of six items (item 1, "concentrate", item 2 "sleep", item 3, "felt useful", item 4, "capable of making decisions", item 7, "enjoy activities", and item 11 "been thinking of yourself as worthless") from the GHQ-12 demonstrated misfit and were subsequently removed from the instrument. The remaining six items (Table 3) comprising four negatively worded (item 5, "felt constantly under strain", item 6, "felt you couldn't overcome your difficulties", item 9, "been feeling unhappy and depressed", item 10, "been losing confidence in yourself") and two positively worded items (item 8, "been able to face up to your problems", and item 12,"been feeling reasonably happy") all demonstrated good fit to the model.

**Table 3:** The fit statistics and item locations for the GHQ-6

Item	Item description	Location	SE	Infit MNSQ	Infit ZSTD	Outfit MNSQ	Outfit ZSTD
**GHQ5**	"Felt constantly under strain"	-0.49	0.04	1.03	1.24	1.04	1.55
**GHQ6**	"Felt you couldn't overcome your difficulties"	0.56	0.04	1.01	0.32	0.98	-0.68
**GHQ8**	"Been able to face up to your problems"	-0.01	0.05	1.11	2.89	1.18	2.90
**GHQ9**	"Been feeling unhappy and depressed"	-0.26	0.04	0.76	-9.55	0.75	-8.72
**GHQ10**	"Been losing confidence in yourself"	0.78	0.04	0.99	-0.18	1.00	0.10
**GHQ12**	"Been feeling reasonably happy"	-0.58	0.05	0.95	-1.52	0.97	-0.63

#### 3. Dimensionality GHQ-12

The principal components analysis of the residuals demonstrated that a six-item scale (GHQ-6) accounted for 70.2% of the variance. The first contrast resulted in two negatively worded items (5 and 6) loading onto one factor, and the other four (two positively and negatively worded items) loading onto the other factor. This contrast in the residuals accounted for only 6.6% of the unexplained variance (eigenvalue = 1.3) suggesting that the GHQ - 6 was unidimensional. However, the subsequent analysis using Smith's method [30] demonstrated that 11% of the paired t-tests fell outside the 95% confidence interval suggesting multidimensionality. It was concluded that although the GHQ-6 was not unidimensional it would still be included in the confirmatory factor analysis.

#### 4. Differential Item Functioning

No differential item functioning (DIF) was observed for gender or treatment aim for the GHQ-6. DIF was observed for a single item (item 8, "been able to face up to your problems") for age. Although there was no difference between the three age groups in terms of the average category endorsed, this item was significantly easier to endorse for the oldest group of patients in comparison with the youngest group (difference = 0.78 logits, t(2803) = 6.26, p < 0.01).

### Rasch Analysis of the GHQ-8

#### 1. Item thresholds GHQ-8

Following on from the Rasch analysis of the GHQ-12 the same Likert scoring system (with collapsed categories for item 11) was applied to the GHQ-8 and item thresholds evaluated. No item threshold disordering was observed.

#### 2. Item Fit GHQ-8

The four items in each of the two factors, Social Dysfunction and Anxiety and Depression (Table 4) demonstrated good fit.

**Table 4:** The fit statistics and item locations for the GHQ-8

Social Dysfunction	Item	Location	SE	Infit MNSQ	Infit ZSTD	Outfit MNSQ	Outfit ZSTD
	**GHQ4**	0.46	0.05	1.09	2.37	1.05	0.69
	**GHQ7**	-1.32	0.04	0.91	-3.01	0.83	-4.95
	**GHQ8**	0.70	0.05	0.97	-0.84	0.82	-2.96
	**GHQ12**	0.16	0.05	0.90	-3.01	0.78	-4.80

**Anxiety/Depression**							

	**GHQ6**	-0.49	0.05	1.19	5.98	1.18	4.59
	**GHQ9**	-1.36	0.04	0.91	-3.19	0.91	-3.09
	**GHQ10**	-0.15	0.04	0.83	-6.33	0.82	-6.22
	**GHQ11**	1.99	0.05	0.96	-0.67	2.38	3.72

#### 3. Dimensionality GHQ-8

An initial PCA was undertaken on the GHQ-8. The first contrast revealed two factors corresponding to the negatively and positively worded items. A subsequent analysis using Smith's [30] method demonstrated that just under 20% of the paired t-test contrasts fell outside the 95% confidence intervals, suggesting the presence of multidimensionality.

Individual PCAs were undertaken for the two factors of the GHQ-8. The principal components analysis of the Social Dysfunction factor demonstrated that this construct accounted for 63.4% of the variance. Furthermore, 14.1% (eigenvalues = 1.6) of the unexplained variance was explained by the first PCA contrast. A similar analysis of the Anxiety and Depression factor revealed that virtually all of the variance was accounted for by this factor (99%).

#### 4. Differential Item Functioning GHQ - 8

No differential item functioning was observed for either factor of the GHQ-8 for any of the subgroup analyses.

### Confirmatory Factor Analysis

The Likert scoring method with collapsed categories for item 11 was used in the Confirmatory Factor Analysis (CFA). The results of the CFA can be seen in Table 5, which demonstrates that the overall goodness-of-fit Chi-square was significant for all six models (similar results were also obtained using the Likert scoring for all 12 items). For the original single factor model, as well as the two factor [21] and three factor models [12] neither the incremental or comparative fit indices (IFI and CFI respectively) reached the 0.95 criterion. The 0.08 criterion for the root mean square error of approximation (RMSEA) was not achieved for the single factor or two factor model (Andrich & van Schoubroeck, [21]) or the GHQ-6 with the 90% confidence interval exceeding this criterion. However, this criterion was met by Graetz's [12] three factor model.

**Table 5:** Confirmatory Factor Analysis of the GHQ - 12

	12 item	12 item*	Model1	Model2	GHQ-6	GHQ-8
**χ^2^**	1608.7	565.55	1018.5	874.71	202.98	265.51
**Df**	54	39	53	51	9	19
**P**	0.0001	0.0001	0.0001	0.0001	0.001	0.0001
**RMSEA **(90% CI)	0.10 (0.097 - 0.11)	0.069 (0.064 - 0.075)	0.081 (0.076 - 0.085)	0.076 (0.072 - 0.08)	0.086 (0.076 - 0.096)	0.068 (0.061 - 0.075)
**ECVI (90% CI)**	0.60 (0.55 - 0.65)	0.24 (0.21 - 0.27)	0.39 (0.35 - 0.43)	0.34 (0.31 - 0.38)	0.081 (0.067 - 0.099)	0.11 (0.095-0.13)
**IFI**	0.89	0.96	0.93	0.89	0.97	0.97
**CFI**	0.89	0.96	0.93	0.94	0.97	0.97

* Correlated error term (Hankins, 2008)

**Model 1 **Andrich & van Schoubroeck (1989) Two-factor model

**Model 2 **Graetz (1991) Three-factor model

The RMSEA criterion was met by both the GHQ-8 and Hankins' [22] unidimensional model with shared error terms, with the former displaying marginally better fit on this criterion. In addition, both of these models also fulfilled the IFI and CFI criteria, as did the GHQ-6. Finally, in terms of the ECVI both the GHQ-6 and the GHQ-8 demonstrated low values for this statistic. Therefore taken together with other statistics it could be concluded that the GHQ-8 had the best model fit of the models evaluated.

## Discussion

The majority of previous studies have demonstrated that the GHQ - 12 is multidimensional and a number of two- and three factor constructs have been proposed. This study aimed to further assess the dimensionality of the GHQ - 12, as well as that of the GHQ - 8 using non-sample dependent tools such as Rasch Models and to evaluate these constructs using confirmatory factor analysis.

The results of the Rasch analysis of the item thresholds demonstrated disordering of thresholds for item 11. Furthermore, these results also revealed a smaller threshold range for negatively worded items suggesting these items were functioning differently.

The Rasch results also confirmed that the GHQ - 12 is not a unidimensional instrument. Six items from the GHQ -12 misfit the Rasch model. Four of these misfitting items corresponded to the putative "Social Dysfunction" subscale [4,18]. Subsequent removal of these items resulted in a six item scale (GHQ - 6) which despite demonstrating good item fit, also exhibited multidimensionality. Although a single item (item 8) was more easily endorsed by the oldest patients no differential item functioning was found for gender and perhaps more importantly treatment aim.

A recent study [18] has suggested an eight item model derived from the GHQ - 12. The Rasch analysis of the GHQ - 8 in this study (using Likert scoring) confirmed the presence of two subscales corresponding to "Social Dysfunction" and "Anxiety and Depression". Both subscales were unidimensional with good item fit and neither subscale demonstrated any differential item functioning.

A comparison of the items from the GHQ - 6 and GHQ - 8 shows some overlap with 5 of the items in the GHQ - 6 also present in the GHQ - 8. The items in the GHQ - 6 reflect both Social Dysfunction ("Been able to face up to problems"; "Feeling reasonably happy"), as well as Anxiety/Depression ("Overcome difficulties"; Unhappy and depressed"; "Losing confidence"), as conceptualised by Kalliath et al. [18]. The three questions included in the GHQ - 8, but not the GHQ - 6 concern decision-making (item 4), enjoying daily activities (item 7) and feelings of worthlessness (item 11).

The results of the confirmatory factor analysis showed that the overall goodness-of-fit chi-squares were significant for each of the seven proposed models. However, Tanaka [41] has suggested that the large sample sizes required to power studies may have the unintended effect of detecting "noninteresting substantive differences" (p. 135), which will affect the concordance between the model and data, and lead to a significant result for the goodness-of-fit. Furthermore, others have stated that stringent assumption associated with this statistic, namely that the model should hold for the population, means that any deviation from this will potentially lead to the model being rejected erroneously [39]. Therefore a comparison of fit indices was undertaken.

The individual indices of fit demonstrated that the incremental and comparative fit indices for Hankins' model [22], the GHQ - 6, and GHQ - 8 exceeded the 0.95 criterion for acceptable models, whereas the other models, including the three factor model [12] fell short of this criterion. For the RMSEA, both the GHQ-8 and Hankins' model [22] demonstrated acceptable fit. The GHQ - 8 had the best overall fit indices, although Hankins' model [22] also demonstrated good overall fit.

Hankins [22] has proposed that negatively worded items introduce additional variance to the model above that created through random measurement error and variations in the measured construct and that this perhaps results from an ambiguous response frame for these items. The results of this study have shown that item variance is indeed greater for negatively worded items than positively worded items, and the results of the Rasch analysis indicate that these items are functioning differently. This study also suggests that response bias to negatively worded items may have a role in explaining some of the multidimensionality observed in previously proposed factor structures for the GHQ-12. However, in terms of comparing the various models the optimum model was shown to be the GHQ - 8 even when accounting for response bias.

These results confirm that the GHQ - 12 is a multidimensional instrument. Furthermore, the study also lent support to the GHQ - 8 proposed by Kalliath et al. [18], and extends this model, which was based on a survey of employees from industrial organisations, in terms of the alternative scoring methods employed, as well as providing support for this model from an alternative sample population, i.e. cancer patients. However, caution should be exercised when interpreting the Anxiety/Depression subscale of the GHQ-8 given that this consists of negatively worded items alone.

A number of studies have found support for Graetz's three factor model [13-17]. However, although the RMSEA fit statistic suggested acceptable fit for this model, both the IFI and CFI fell below the minimum criterion. These results replicate the findings of others [18] that when considering a number of fit indices there is less support for the three factor model proposed by Graetz [12].

The study is potentially limited by the fact that the sample was drawn from a cancer population where the majority of patients (>60%) were female and in late middle age. Nevertheless this should be balanced against the fact that a large sample size was utilised in the study.

Some authors have recommended continuing to use a summary index of the GHQ-12 despite the presence of multidimensionality, due to the high degree of inter-item correlation [14], however given the level of potential confounding variables, such as misfit, multidimensionality, and item variance found in this study this practice could potentially lead to an erroneous assessment of patients' psychiatric morbidity.

## Conclusion

This study provides further evidence that the GHQ-12 is a multidimensional instrument. Although negatively worded items demonstrated greater variance, when this was accounted for an eight-item version of the GHQ12 (with two factors: Anxiety/Depression and Social Dysfunction) displayed the best model fit in a comparison of factor structure models. Further study into the factor structure of the GHQ-12 is warranted for different target populations.

## Competing interests

The authors declare that they have no competing interests.

## Authors' contributions

ABS undertook the analysis of the questionnaire. ABS, LJF, DS, GV and VJ all contributed to the drafting of the manuscript. All authors have read and approved the final manuscript.
